# Exercise-Induced Asthma Symptoms and Nighttime Asthma: Are They Similar to AHR?

**DOI:** 10.1155/2009/378245

**Published:** 2009-11-12

**Authors:** V. Backer, L. M. Rasmussen

**Affiliations:** Department of Respiratory Medicine, Bispebjerg Hospital, University of Copenhagen, 2400 Copenhagen NV, Denmark

## Abstract

*Background*. Asthma experienced during exercise and during the night is based on the presence of airway hyperresponsiveness (AHR). The aim of the present study was to examine whether AHR is a predictor of exercise-induced asthma (EIA) and nighttime symptoms. *Material*. We included 793 asthmatics subjects with symptoms and a positive asthma test. *Results*. Mean (SD) FEV1 was 93% (15), 71% had rhinitis, and 62% had atopy. Both EIA and nighttime symptoms were associated with AHR; however, when including other factors of importance in a multivariate analysis, logRDR was eliminated, whereas FEV1% pred (*P* < .001), smoking (*P* < .05), atopy (*P* < .001), sex (*P* < .001), and treatment (*P* < .01) were associated with having EIA while dyspnoea (*P* < .001), cough (*P* < .001), and eosinophils (*P* < .01) were associated with frequent night symptoms. The risk of having nighttime awakenings due to asthma was more than twofold higher among those with EIA symptoms than among those without symptoms (OR (CI95%) 2.77 (2.0–3.8) (*P* < .001)). *In Conclusion*. EIA and night symptoms are associated with AHR, but other factors of importance eliminated this close association. Night asthma is more closely associated with airway inflammation than AHR.

## 1. Introduction

Asthma severity and asthma control are often based on the presence and frequency of asthma symptoms, in particular respiratory symptoms such as exercise-induced asthma, limitation of physical activity, and frequency of night asthma symptoms. The revised version of the GINA guidelines from 2006 [1] recommends that disease classification can be changed from severity based on symptoms and airflow limitations to success of disease management. Disease management is categorised as controlled, partly controlled, and uncontrolled asthma. This is mainly based on frequency of symptoms and continues to include limitation of physical activity and night symptoms suggestive of level of airway inflammation and increasing need of inhaled corticosteroid (ICS). 

Perception of respiratory symptoms may vary between individuals—some might feel minor changes more than others [2]. Physical activity is an important release of asthma symptoms, revealing exercise-induced bronchoconstriction [3, 4], which is widely believed to be related to either airway inflammation [3] or smooth muscle dysfunction [5–8]. The primary treatment strategy in airway inflammation should be inhaled steroid [9]; whereas smooth muscle dysfunction should be treated primarily with inhaled beta-agonist [6]. There are data to support both suggestions, but the treatment management differs. Nighttime awakenings are probably related to increased airway inflammation [10, 11]; however, treatment strategies including lower dose of ICS combined with long-acting beta2-agonist have been found to induce a more stable disease than a higher dose of ICS alone, which is contradictory to increased inflammatory control [12]. EIA and night asthma might arise from airway inflammation, but other factors, such as smooth muscle dysfunction or differences in the patient's perception/tolerance of symptoms, might play a role. The aim of the present study was to examine in a large population sample whether AHR is of importance for the perception of EIA, that is, positive response to a questionnaire and nighttime symptoms. The hypothesis is that symptoms of EIA in a general population are not usable for the diagnosis of astma.

## 2. Material and Methods

### 2.1. Study Population

All asthmatic subjects (*n* = 793) participating in two large asthma studies in our research unit were pooled in the present analysis [13, 14]; inclusion and exclusion criteria and the examinations performed were the same in all studies. Subjects were aged 14–44 years (to minimize entry of patients with COPD) and all lived in Copenhagen, Denmark. Exclusion criteria were respiratory illness other than asthma, for example, rhinitis as single disease, sarcoidosis, and cardiac illness. Patients were excluded if they had withdrawn their consent after completing the questionnaires or if they had left the clinic without completing the clinical examination or diagnostic procedures. The studies were approved by the local ethics committee of Copenhagen, Denmark. All participants received information in both oral and written form and gave their consent in writing before enrolment.

### 2.2. Study Design

#### 2.2.1. Medical History

All participants completed five self-administered questionnaires before clinical and physical tests. Subjects were asked about respiratory and allergic symptoms (within the preceding four weeks and at any time (ever asthma)), use of medication, hospital referrals, and GP or specialist visits. The questions asked about asthma at the interview were adapted from studies by the American Thoracic Society, Division of Lung Disease of the National Heart, Lung and Blood Institute [15]. Asthma was defined as asthma symptoms and signs of reversible airway disease, that is, either airway hyperresponsiveness (AHR) to inhaled methacholine with a PD20 ≥ 4 *μ*mol, peak expiratory flow (PEF) variability ≥ 20%, or at least a 15% increase in forced expiratory volume in one second (FEV1) after administration of a bronchodilator (minimum 300 mL).

### 2.3. Definition

Asthma severity was classified according to the GINA guidelines (1) *Mild intermittent*: symptoms <once a week, nighttime symptoms <twice a month, FEV1 > 80% predicted. (2)* Mild persistent*: symptoms >once a week but <once a day, nighttime symptoms >twice a month, FEV1 > 80% predicted. (3)* Moderate persistent*: daily symptoms, nighttime symptoms > once a week or FEV1 60–80% predicted. (4)* Severe*: continuous daytime symptoms, frequent nighttime symptoms or FEV1< 60% predicted. Further, nighttime awakenings “0” (never), nighttime awakenings (NTA) ≤ 3 per month (mild), NTA 3-4 per month (mild persistent), NTA >4 per months and fewer than 2-3 per week (moderate), and NTA > 4 per week (severe). The severity of asthma (mild, mild persistent, moderate, and severe) was classified according to the 2004 GINA guidelines, based on symptom frequency only [16], and asthma control was assessed according to the definition in the 2006 GINA guidelines [1]. Details about education, socioeconomic aspects, and lifetime consumption of tobacco in pack years (tobacco consumption [g/day]/20 ∗ duration of smoking [years]) were collected. Never smokers were participants who have never smoked, whereas smokers included daily smokers and smokers with a more dispersed consumption. Height and weight were measured and body mass index (BMI) was calculated as (weight [kg])/(height [m]^2^).

### 2.4. Methods

#### 2.4.1. Pulmonary Function Test and Methacholine Challenge Test

Spirometry was performed on a 7-L dry wedge spirometer (Vitalograph) in accordance with the ERS and the percentage of predicted normal values of FEV1 (FEV1%pred) and FVC (FVC%pred), and the FEV1/FVC ratio was calculated [17]. Airway responsiveness to inhaled methacholine was measured in accordance with the method of Yan et al. [18] in all patients with FEV_1 _>70% predicted. The dose resulting in a 20% fall in FEV_1_ (PD_20_) was calculated, and the ratio dose response (RDR) was calculated as the decline in FEV_1_ from inhaled saline divided by the highest dose of methacholine administered [19]. A constant of four was added to all dose-response ratios to eliminate negative and zero values. Logarithmic transformed values of RDR were used for analysis. Measurement of FEV_1_ was repeated 15 minutes after administration of 2 mg terbutaline in those with FEV1 < 70% or 15 minutes after the last inhalation of methacholine in those with either symptoms or a significant decrease in FEV1 (i.e., 20%).

#### 2.4.2. Allergy and Inflammatory Testing

Lastly, all subjects underwent an allergen skin prick test with ten inhalant allergens in accordance with the EAACI guidelines [20], and blood eosinophils were counted (10^9^/L).

### 2.5. Analysis

All data were entered in the database by one person and 10% of all patient data were then proofread. When this was done, quality control was conducted by an experienced researcher on all outliers within the entire number of variables. 

The data were analyzed with the statistical pack 15.0 SPSS for Windows. Incidence rates were calculated for the entire group and differences were tested by Chi-squared analysis. Further, differences in mean (±SD) values between participants with and without respiratory symptoms were tested by parametric analysis (Student's *t*-test). All included variables were examined in a univariate analysis with exercise-induced asthma or nighttime awakenings due to asthma as the dependent variable. In the event of a significant relationship, variables were entered in a linear regression analysis with backward elimination of all nonsignificant parameters after which a final regression analysis was performed. Lastly, an odds ratio analysis was applied in the case of dichotomy variables. A *P*-value <.05 was considered significant.

## 3. Results

### 3.1. Basic Parameters

All 793 asthmatic subjects in the present survey were included based on respiratory symptoms and a positive test to methacholine provocation, inhaled beta-agonist or day-to-day variation in PEF (Table 1). The frequency of rhinitis was 71% among those with certain asthma, 62% had positive skin prick test, and 55% were never smokers. The frequency of exercise-induced asthma was as follows: never symptoms 9%; 54% ≤ 2 times per week; 22% 2–6 times per week; 7% daily exercise symptoms; 8% reported EIA more than once a day. The night awakening due to asthma was 69%, 12%, 7%, 6%, and 6%, respectively. Of the entire group, 29 asthmatic subjects (3.1%) reported both severe EIA and nighttime awakenings, and 55% reported neither symptom (*P* < .001).

### 3.2. Univariate Analysis of EIA and Nighttime Awakenings

A univariate analysis including severity of EIA (Table 2) and nighttime awakenings (Table 3) showed a significant association between airway inflammation, indicated by responsiveness to inhaled methacholine (logRDR), and blood eosinophil count (10^9^/L), or obstruction and severity of symptoms. The association between airway responsiveness and EIA symptoms (F = 2.5, *P* < .05, and rho = 0.1, *P* = .01) was less close than that between airway responsiveness and nighttime awakenings due to asthma (F = 3.5, *P* < .01 and rho = 0.14, *P* < .001). Further, no significant association was found between rhinitis and severe EIA (15.4% versus 15.1%, NS) and nighttime awakenings (11.9% versus 10.9%, NS); atopic diseases was seldom seen in those with severe EIA (10.0% versus 14.3%, *P* = .08) or among those with severe night symptoms (12.4% versus 21.0%, *P* < .01). Experience of severe EIA was frequently found among female participants (21.0% versus 7.3%, *P* < .01), and nighttime awakenings were found equally frequently among those who had severe EIA (12.9% versus 8.5%, resp., NS). Lastly, those with severe EIA also had many nighttime awakenings (56.8% versus 11.4%, *P* < .001); those with many nighttime awakenings also experienced many symptoms of EIA (24.0% versus 4.8%, *P* < .001).

### 3.3. Multivariate Analysis of EIA and Nighttime Awakenings

Concerning EIA, by including all variables in a multivariate analysis, logRDR was eliminated; whereas FEV1%pred (*β*−.16, *P* < .001), smoking (*β* .098, *P* < .05), atopy (*β*−.16, *P* < .001), sex (*β*−.18, *P* < .001), and asthma treatment (*β* .17, *P* < .01) were found to be of significant consequence for development of EIA. Women reported persistent exercise symptoms more frequently than did men (21% versus 7%, *P* < .001); smokers had more EIA than nonsmokers (18% versus 13%, *P* < .05); BMI was of no importance. Asthmatic subjects with persistent exercise symptoms had lower lung function than those without symptoms (88% versus 94%, *P* < .001), and treatment with inhaled steroid was more frequently used among those with persistent exercise symptoms (41% versus 17%, *P* < .001).

Including all variables in a multivariate analysis concerning nighttime awakenings showed that logRDR was eliminated as well; whereas frequency of shortness of breath during daytime (*β* 0.263, *P* < .001), coughing (*β* 0.243, *P* < .001), and EIA (*β* 0.102, *P* = .066) were of significant consequence. Furthermore, a higher level of eosinophils was associated with a higher level of nighttime awakenings (*β* 0.155, *P* < .011). These findings showed that among those with frequent night symptoms, 64% experienced daily coughing, 31% had daily dyspnoea, and the eosinophil count increased from 0.22 10^9^/L (no night symptoms) to 0.35 10^9^/L (frequent night symptoms), (normal cell count 0-0.4 10^9^/L). 

The relative risk of having nighttime awakenings due to asthma was more than twofold higher among those with EIA symptoms than among those without EIA symptoms. In addition, the odds ratio (CI95%) was 2.77 (2.0–3.8) (*P* < .001) of having nighttime awakenings due to asthma among those with EIA symptoms.

## 4. Discussion

Although many different surrogate variables representing airway inflammation—such as bronchial responsiveness, low level of lung function, signs of airway obstruction (low FEV1/FVC ratio), positive skin prick test, high eosinophilic cell count, and many daily asthma symptoms—were included in the analysis of this large uniform cohort of persons with asthma, the study showed great diversity. EIA and nighttime awakenings due to asthma symptoms are symptoms of equal importance in the GINA guidelines [21] when evaluating the severity of asthma. According to the present guidelines, severity of asthma is based equally on the frequency of respiratory symptoms, such as exercise-induced asthma symptoms, and nighttime awakenings due to asthma symptoms [21]. Less controlled disease is usually an indicator of increased airway inflammation, and the guidelines therefore suggest initiation of inhaled steroid. From a theoretical viewpoint, exercise-induced bronchoconstriction could occur only in the event of airway inflammation, and the more severe the EIA the more severe the airway inflammation that could probably be demonstrated [3–5, 11, 22]. Symptoms of EIA in elite athletes are thus not to be used as a predictor for asthma [23, 24] as those symptoms are not related to either airway hyperresponsiveness to inhaled agents or to indirect provocation. It is thus unknown whether EIA symptoms are closely associated with airway responsiveness and airway inflammation in a general population of asthmatic subjects who have demonstrated certain airway variability, because the majority of studies have been performed in groups with selected asthmatic subjects who report EIA. However, the present study showed that factors important for the presence of EIA symptoms and nighttime awakenings were outside the battery of inflammatory variables.

These present findings indicate a close univariate association between EIA symptoms and AHR, which is in agreement with earlier findings of a close association between degree of EIA and AHR to inhaled agents [25, 26]. However, in the final analysis many inflammatory variables were statistically eliminated, whereas EIA symptoms continued to show a significant association with low level of lung function and positive skin prick test, which could suggest an association between markers of airway inflammation and EIA in asthmatic subjects with a history of EIA symptoms. One of which, the most important predictor of airway inflammation in the present study, may be having low level of lung function, as low level of lung function could be due to inflammatory swelling of the airway mucosa [27–29]. 

The asthmatic women frequently reported persistent exercise-induced symptoms; whereas nighttime awakenings in asthmatic participants in this large population were without sex association when interview by a specialist using a questionnaire-based interview with focus on the issue, as suggested by GINA guidelines [1]. We found this sex difference interesting. It indicated that women may have a lower tolerance to EIA or perhaps a different perception of EIA compared with that of men. An explanation of women's lower tolerance to EIA could be a symptom of low fitness, and not astma or airway inflammation. Women probably perform fewer physical activities than do men, especially among the oldest in this group; consequently, when they exercise they develop more symptoms. Another explanation could be the level of lung function, because although both women and men who reported severe EIA generally had low level lung function, the women had a significantly larger reduction in FEV1 percentage predicted than did the men (data not shown), indicating that low ventilatory capacity could also be a satisfactory explanation of the present results of the EIA symptoms. Furthermore, we found that more smokers than nonsmokers claimed exercise symptoms. Other studies indicate that asthmatic persons who are smokers have impaired effect of their asthma treatment, and compared with the asthmatic subjects who were non-smokers, the asthmatic smokers showed low level of lung function, less improvement in lung function, BHR, and symptoms [30, 31]. These findings support our finding of more EIA symptoms among those asthmatic subjects who smoked, although it is unexplained whether it is due to the smoking history or the low level of lung function, but it seems not to be inflammation. 

Those asthmatic subjects with severe nighttime awakenings seem to have more severe illness than those with EIA symptoms. Those with night symptoms had a high eosinophilic count; whereas the perception of exercise-induced symptoms had a cell count which was significantly lower. Those with nighttime awakenings due to asthma had a more severe degree of airway responsiveness than did those with exercise symptoms; moreover, those who had severe night awakening also had frequently signs of other asthma symptoms, such as dyspnoea and cough during daytime, all of which indicate that those with nighttime awakenings due to asthma might have severe airway inflammation and need treatment with inhaled steroid. These findings indicate that symptoms of EIA may be less related to inflammation, whereas nighttime awakenings are more closely related to airway inflammation. The higher eosinophilic count found among those with many night awakenings is of significant importance as the prognosis of asthmatic subjects with high level of eosinophilic cell count; many respiratory symptoms and severe AHR have been shown to be serious with a more severe and more variable asthmatic disease [32]. Nighttime awakenings due to asthma compared with EIA symptoms without objective measurements are probably more likely to be based on airway inflammation, which indicates that these asthmatic subjects with nighttime awakenings, but not symptoms of EIA, would probably benefit from increased treatment with inhaled steroid [32–34]. However, we found a significantly increased risk of having nighttime awakenings among those with exercise-induced asthma symptoms. The risk was almost threefold increased among those with many EIA symptoms and nighttime symptoms, indicating some association between the two different questions of asthma symptoms, and that although the nighttime awakenings seem to be more closely associated with airway inflammation, the presence of many exercise symptoms also has some relation to the presence of airway inflammation, but the majority of symptoms might be due to low level of fitness.

Based on these findings and earlier findings in elite athletes [23, 24] the GINA guidelines would most likely benefit from reducing the importance of EIA symptoms as an initiator of increased treatment strategy of inhaled steroid without having an exercise test showing EIB. These findings also emphasise the importance of objective measurement of asthma, including objective measurement of EIA symptoms, before including EIA as important for the level of asthma severity and the level of control. It could be speculated, however, whether some of these symptoms are based on a smooth airway dysfunction rather than inflammation or low level of fitness, indicating need of bronchodilator and not steroid, as all participants had proven asthma. In the present study a substantial number of asthmatic subjects were either untreated or undertreated [13]; this has been reported in earlier publications. These findings of undertreatment in the present population of asthmatic subjects highlight the importance of the present findings. The subjects were not contaminated with inhaled steroid, and even those who reported serious night awakening due to asthma were not receiving treatment according to the guidelines. 

*In conclusion*, in this large group of asthmatic subjects we found that EIA symptoms and night symptoms were associated with AHR in a univariat analysis, but other factors seem of importance for the perception of EIA and night symptoms. Symptoms of EIA in a general population are, like EIA symptoms in elite athletes, not usable for the diagnosis of astma. Night asthma is more closely associated with airway inflammation, which verifies what we had expected.

## Figures and Tables

**Table 1:** Basic variables (mean and SD) of the entire group of asthmatic subjects.

Variables	Mean (SD)
Sex (females)	61%
Rhinitis (%)	71%
Age (years)	29. (8.3)
BMI (m^2^/kg)	25. (10.9)
FEV1 (L)	3.5 (0.8)
%pred FEV1	92.7 (14.9)
FVC (L)	4.33 (1.0)
%pred FVC	95.3 (13.1)
FEV1/FVC%	81.2 (8.3)
logRDR Δ%FEV1/*μ*mol	1.02 (0.5)
Eosinophil counts (10^9^/L)	0.24 (0.2)
Pack year (cigarettes/day)	8.21 (8.0)

**Table 2:** Basic variables and association with EIA symptoms in an univariate analysis.

	EIA 0	EIA 1	EIA 2	EIA 3	EIA 4	EIA all	*P*-values
BMI	24 (4)	25 (4)	25 (5)	27 (7)	26 (6)	25 (5)	*P* < .001
Age	30 (6)	29 (8)	29 (9)	29 (8)	30 (9)	29 (8)	NS
%FEV1	94 (13)	94 (14)	92 (14)	92 (14)	84 (19)	92 (15)	*P* < .001
%FVC	96 (10)	97 (13)	94 (13)	95 (13)	89 (14)	95 (13)	*P* < .001
FEV1/FVC%	81 (7)	82 (8)	81 (8)	81 (7)	79 (12)	81 (8)	*P* < .001
logRDR	0.95 (0.4)	0.99 (0.4)	1.06 (0.5)	1.17 (0.6)	1.03 (0.5)	1.02 (0.5)	*P* < .05
Eosinophils	0.23 (0.2)	0.23 (0.1)	0.25 (0.2)	0.28 (0.3)	0.20 (0.2)	0.24 (0.2)	NS
Pack years	6.8 (5)	7.8 (7)	8.4 (8)	10.2 (9)	10.0 (11)	8.3 (8)	NS

EIA 0 never, EIA 1 ≤ 2 per week, EIA 3 2–6 per week, EIA 3 daily, EIA 4 more than daily.

**Table 3:** Basic variables and association with night awakening due to asthma symptoms in a univariate analysis.

	Night 0	Night 1	Night 2	Night 3	Night 4	Night all	*P*-values
BMI	25 (5)	25 (4)	25 (5)	27 (7)	25 (5)	25 (5)	*P* < .01
Age	29 (8)	28 (8)	29 (8)	31 (8)	34 (7)	29 (8)	*P* < .001
%FEV1	94 (14)	92 (14)	88 (11)	93 (14)	82 (19)	92 (15)	*P* < .001
%FVC	96 (13)	96 (13)	91 (15)	97 (13)	89 (15)	95 (17)	*P* < .001
FEV1/FVC%	82 (8)	80 (8)	81 (8)	80 (8)	75 (12)	81 (8)	*P* < .001
logRDR	0.99 (0.4)	1.03 (0.4)	1.19 (0.5)	1.11 (0.6)	1.13 (0.6)	1.02 (0.5)	*P* < .01
Eosinophils (10^9^/L)	0.22 (0.1)	0.25 (0.1)	0.27 (0.2)	0.30 (0.3)	0.35 (0.3)	0.24 (0.2)	*P* < .01
Pack years	7.7 (7)	6.9 (6)	6.0 (6)	11.3 (9)	14.8 (12)	8.2 (8)	*P* < .001

Night 0 never, Night ≤ 3 per month, Night 3–4 per month, Night >4 per month and fewer 2-3 per week, Night 4 > 4 per week.
